# Etiology, Pathology and Treatment of Pellagra; Cotton Seed Oil and Animals

**Published:** 1911-08

**Authors:** George C. Mizell

**Affiliations:** Atlanta, Ga.; Formerly Associate Professor of Physiology and Gastro-Enterology of Atlanta College of P. & S.; Formerly Gastro-Enterologist to Wesley Hospital


### ETIOLOGY, PATHOLOGY AND TREATMENT OF PELLAGRA; COTTON SEED OIL AND ANIMALS.

By George C. Mizell, M.D., Atlanta, Ga.

*Formerly Associate Professor of Physiology and Gastro-Enterology of Atlanta College of P. & S.; Formerly Gastro-Enterologist to Wesley Hospital.*

In the preliminary report on the cause and treatment of Pellagra published in this magazine last month, I endeavored to give an outline of the facts and operation of the cause in the production of the disease. I now propose to go fully into detail and give data which will show more conclusively that the theory is well founded and remains only to be verified by the results when the use of cotton seed oil as a food is discontinued in this country.

First. I will go fully into the nature of the disease ac-according to this theory. When the nature is understood we have a complete explanation of the various symptoms. Observation of the symptoms indicates that there are two forms, acute and chronic. It appears that the form is in part determined by amount and regularity with which Linolin is taken into the body. In some cases it seems to accumulate by deposit in the tissues with little evidence of disturbance, then, under the influence of some secondary condition there is an outbreak which is violent and runs a rapid course. In other cases the beginning of the use of cotton seed oil products is accompanied by symptoms which are more or less mild and slow in progress. The course of the disease, I believe, is determined by conditions which are in evidence in any large collection of cases.

The conditions which favor an acute course are:

Tendency to accumulate fat. Weak digestive apparatus. Acute illness. Child bearing in women. Eating oils containing a large per cent. of Linolin. (Cotton seed oil from which the stearin has been removed contains a very large per cent. of Linolin.) Certain drugs which promote oxidation. Drugs forming aldehydic substances when introduced into the body. Exposure to the light and heat of the sun.

The conditions favoring a chronic course are:

Tendency to use up the fat as soon as taken into the body. Those who are ematiated when the consumption of cotton seed oil begins and who do not accumulate the fat. Eating oils that contain a small per cent. of iLnolin. Limited exposure to the light and heat of the sun. Strong digestive apparatus.

People who are strong and vigorous may eat semi-drying oils for years and in limited amounts for a long time and manifest no deleterious effects until something disturbs the digestion and forces them to reduce the amount of food taken. During this period of partial starvation the reserve fat is called upon and we have an acute progress started. People who live in the city are not, as a rule, as much exposed to the sun, hence, are not near so liable to develop skin symptoms. A weak digestion facilitates development of the acute form by being more easily damaged by the oxidation products. When the digestion is crippled the food has to be limited and the reserve tissues are called on. The oxidation of Linolin as of all body fat is retarded by a plentiful supply of nourishment introduced into the blood.

It is necessary to bear in mind that the symptom complex may be developed at any time during the consumption of semidrying oils and that great damage may be in progress during this period. It appears that the greatest damage is done to the secretory apparatus of inorganic agents first and then of the organic agents of the digestive system. Hydrochloric acid is first suppressed, then rennin and pepsin. (Later we will give results of examinations which show this to be the case.) It is only mentioned here in explanation of delayed symptoms of damage done. Much information can be gained by a study of the tabulated report of cases by Dr. Johnson.

Every one who makes a practice of examination of gastric contents in chronic disease knows that a complete absence of these digestive agents in a great many cases is compatible with seemingly good health. However, this state of well-being does not last long on a promiscuous diet without manifesting itself. So it is with consumers of semi-drying oils. There may be great damage done without attracting the attention by symptoms, but this cannot exist for any great length of time. I do not agree with those who claim that pellagra is always a slow, chronic process, inflicting damage over a long period of years before developing the classic clinical picture. Several years ago, before the recognition of the disease by the laity, few of the mild cases consulted a physician, or the physician treated these cases as sunburn, erysipelas, eczema, tetter, etc. Since the disease is known to the laity and general practitioner, we are seeing many cases who have suffered with no symptoms except the erythema for several years.

Some of our writers are disposed to believe that the lesions of the central nervous system are first and chief organs affected. As will be shown later clinical and post-mortem evidence excludes this view. Below is given a few histories which show both forms of the diseases. These cases are not selected, except one, but are consecutive, all having presented themselves for trsatment in the last few weeks. Every one of our cases have eaten cotton seed oil. The shortest period from the beginning of the use of cotton seed oil to the appearance of the erythema, is eight months. The longest period of continuous use without symptoms is six years. There are many cases of "Pellagra sine Pellagra" not contained in these records and will be referred to later.

Case 301, Mr. C., Eating compound lard one year.

Past History. Indigestion two years. Bowels loose six weeks, but some better now. Four months ago had erythema on hands, which disappeared in four weeks to return in six weeks.

Present symptoms. Three loose stools, containing blood and mucus, daily. Cramp in bowels daily. Nervous. Symmetrical eruption on hands. Feet and ankles swollen.

Case 341, Mr. C., Cotton seed oil three, four or five months in each year for five years.

Past history. Good health till two years ago, when developed erythema on hands.

Lost fifty pounds in eighteen months. Normal weight, 185 pounds.

Present symptoms. Weak, nervous, doesn't sleep but few hours daily. Thick, ropy saliva. Mouth sore. Two loose, watery stools daily.

Note that Mr. C., who was a farmer, ate oil products three, four or five months annually. This always took place in the fall. Until this illness he enjoyed good health.

Case 449, Mr. M.—Presented himself for treatment April 1, 1911.

Using cotton seed oil products one year.

Past history. Good health. Some indigestion five or six years. Present illness began about Dec. 25th. 1910.

First symptoms were too much appetite and heaviness in stomach after meals. Headache in occipital region at night. Complains of burning over right blade shoulder. Lost five pounds in six months. Symmetrical erythema on backs of hands which appeared in January, 1911. Very nervous and dizzy. Sleeps poorly. Burning in stomach.

Present symptoms. Above symptoms are still present. Patient is constipated, has marked mental symptoms and complains of the irresistible fear that he would do violence to his family.

This case was selected, being of especial interest as to the date of the eruption, which occurred in January. An explanation of this is to be found in the fact that during December, which was rather warm for the season, he worked on the roof of a house and his hands were exposed to the sun.

This man was in good health until this period of exposure and the unusual physical exercise in putting shingles on the roof of a house. His occupation was stationary engineer, and he heretofore took very little exercise, all the time protected from the sun.

Case 329. Mrs. H.—Age 37. Weight 140 pounds. Using compound lard off and on twelve to fifteen years.

Past history. Has had erythema for four years, but general health was good till one month ago.

Present history. Last February hands, face and arms were severely broken out, recovered and doing well till six weeks ago, when it returned and has suffered with indigestion. Bowels are loose. Losing weight. Appetite poor. Dizzy. Some nausea and vomiting. Mouth sore three weeks. Ankles and limbs swollen and pit on pressure. Very nervous. Sleep varies. Has redness, tenderness and itching around vulva and rectum. Has slight mental symptoms.

Important points in this case are good general health even after reappearance of the erythema for four years. Only after the second recurrence in this year of the erythema and the appearance of severe gastric and intestinal symptoms has she felt unwell.

Case 694, Mrs. M.—Age 31. Weight 103 pounds. Using lard compound five years.

Past history. Bad health five years. Constipation and diarrhoea three years. Erythema two years.

Present symptoms. Strange feeling in eyes. Feet and hands burn. Burning in chest and has attacks of colic. Has attacks of diarrhoea which last a few days. Has bladder trouble and irregular female trouble.

Compare this case with case 329. Case 694 has always been light weight, while 329 was inclined to be fat. In one the digestive symptoms began with the use of lard compound, in the other the digestion was apparently good after twelve to fifteen years' intermittent use. 329 is in an acute attack after a slow accumulation of 12 to 15 years.

Case 574, Mr. D.—Age 38. Farmer. Used compound lard four months annually for five years.

Past history. Has continued in good general health and at work to date. Has had no inconvenience. For three years bowels have been loose at times. Erythema on hands one year ago and reappeared on May 10, 1911. Has had two to five loose stools daily for two months.

This case presents no symptoms outside of the ones above mentioned. It was a well marked case of the disease without any derangement of the nervous system.

Case 680, Mrs. H.—Age 51. Using lard compound two years.

Past history. Two years ago had operation for fibroid tumor in uterus. Had erythema, accompanied by stomach trouble and loss of strength, in spring of 1910 and again in 1911. Patient is well nourished.

Present symptoms. Hands and feet burn and itch. Erythema severe and extends into the palmer surface of the hands. No appetite, and nauseated. Sleeps poorly. Vertigo and staggers when walks. Usually constipated. Attack of diarrhoea last week. Has sour stomach and burning in chest and stomach. Stomach and bowels sore. Head feels strange.

Patieint is in an acute attack. Food has to be forced and is often vomited. Mouth is covered with white patches. Tongue very red and shows marked indentations of teeth. Salivation is extreme. This belongs to a class of cases that usually is rapidly fatal.

Case 639, Mrs. J.—Age 39. Weight 170 lbs. and gaining. Using a cotton seed oil product seven years.

Past history. Had malaria fifteen years ago and health has been bad since. Two weeks ago had chills and fever. Constant aching in epigastrium, also pain in left iliac. Pains in back and arms for years. Stomach trouble six years. Erythema in spring of 1910. Complains of fatigue during warm weather. Eats general diet.

Present symptoms. Patient has noticed that the least exposure to the sun makes hands red and wears gloves for protection. If she wears a thin waist the neck and shoulders "blister." The hands now show a slight erythema.

Appetite is fine. Has fullness after meals and pain in pit of stomach. Has sour stomach, burning and some soreness in stomach. Flatuency in bad and has palpitation. Nervous at times. Legs below knees dent on pressure—ankles swell.

This is a very interesting case. The patient states that she only weighed 105 pounds when she began using cotton seed oil products. She weight 170 now and notwithstanding malaria (microscope shows plasmodium) she is still gaining. This gain in weight by those who have begun eating cotton seed oil is not unusual. It is consistent with the easy digestibility mentioned below. Because of this tendency to add to the body weight it has gained great popularity in treating tuberculosis.

Case 402, Mrs. B.—Weight 115 pounds. Cotton seed lard nine months.

Past history. Bad health for eight years from ovarian trouble. Eruption on hands five weeks.

Present symptoms. Slight erythema on hands. Feet ache. Lost 30 pounds since Jan. 1, 1911. Nervous at times. Nausea occasionally. Bowels regular. Appetite good.

Notice that this patient weighed 145 when she began eating lard compound and began losing weight three months thereafter. Her symptoms are slight as compared with case 639. Such is pellagra. Individual factors that are as numerous as individuals themselves play the all-important part in directing the course of results from eating these seed oils.

It is consistent with this theory to believe that a number of months may elapse after discontinuing the use of semi-drying oil before the danger of developing symptoms is passed. How long a certain area of fat will remain in the body is in part governed by the amount and character of the food eaten. It may be true that a remnant may remain for an indefinite period. We saw a severe case develop six months after the exclusion of these products from the diet. The disease may develop at any time so long as semi-drying oil is present in the tissues. If erythema appears in the autumn and is for any reason arrested before all of the Linolin is oxidized, it necessarily follows that it may recur next spring if it is not removed during the winter, though the consumption of oil is discontinued.

None of our cases whose diet excluded cotton seed oil have recurred after two seasons, but we have had several cases in which the erythema disappeared in the early spring to recur to a slight degree in one or two months. In these cases the area affected was most marked n those parts not previously affected.

Ths behavior of the erythema, so far as I know, has not been mentioned. It further bears out this theory in that it shows that the conditions attending the erythema are no other than the character of the tissue and the action of the heat and light of the sun. A careful study of the conditions concerned in the production of this one characteristic symptom will aid in a more clear application of this theory and understanding of the disease. In America we know that the parts of the body affected by erythema are those exposed to the sun. In Roumania, where the gypsy children go naked in the street, we have a demonstration that the erythema occurs on other parts of the body but most marked on the extremities. This shows that the agent in the tissues is not equally distributed over the body. Bearing on this difference in the tissue, we have the statement of Lewkowitsch who says that the liquid fats diminish in proportion as the fat approaches the warmer parts of the body. As Linolin is one of the most fluid fats, it would be deposited in the extremities to the greatest extent. Two cases reported above are of interest in showing the fixed nature of this agent in the tissues. In one case after about a month's confinement to the house a severe erythema had cleared up and the patient was free from symptoms until he took an automobile ride during the heat of the day. In a few days the erythema reappeared, but this time affected the portion of the skin surrounding the site previously affected. The constitutional symptoms also reappeared and persisted a few days.

The other case presented a mild erythema affecting the entire dorsal surface of the hands. This disappeared and the hands were smooth after a month of confinement to the house. A month later, as a matter of experiment, he was told to go out in the sun. In a few days there appeared at the site of the previous erythema a redness, which later faded out and was followed by the exfoliation.

It has been stated that the palmer surfaces are not affected, but this is shown to be an error by two cases recently under our observation. One, a child, age seven, where the process was so severe that there was a cast of both hands. So complete was this cast that it could have been removed like a glove, had not the skin been cut away as it separated from the underlying thin, delicate epidermis. This was an extreme case of the so-called moist erythema. A correct interpretation shows moist erythema to be due to application of certain remedies, or infection of abrasions in the delicate epitbelium covering those portions of the affected part from which the embalmed superficial skin has separated.

The history of pellagra in this country is not at all complete. The extent is appalling to the laity and the medical profession. Physicians are beginning to recognize "pellagra sine pellagra." We have seen about 200 cases and many other physicians in this state have seen the disease in similar numbers. In one small city of 5,000 inhabitants one physician of three has twenty-five cases. In an orphans' home there were fifteen cases. It is conservative to accept the statement of an official of the State Board of Health that there are 50,000 cases in the South. Reports from South Carolina and Alabama show about same prevalence of the disease. Without statistics we must rely on our individual experience. This experience places the beginning of the period of relatively numerous cases at about 1908.

Sporadic and imported cases have occurred for a number of years. In order to show that the history of Pellagra in the United States parallels the consumption of cotton seed oil in America, I give the following information from a reprint from Year Book of Department of Agriculture for 1903.

"That none of the exclusively oil-yielding seeds have ever been cultivated in the United States for oil-making purposes has been due primarily to a lack of demand for their products. A distinctive characteristic of the American people, though modified in recent years, has been the use of animal fats, both in domestic and industrial life, for many purposes for which the inhabitants of other countries largely employ vegetable oils. In domestic life there has always been in the mind of the American housewife a somewhat inexplicable prejudice against the use of vegetable oil for cooking purposes, and until recent years lard has completely usurped the functions here that from remote antiquity has been accorded in many countries to vegetable oils. That this prejudice is being gradually mollified there is no doubt, but it is a tribute to its persistency that vegetable cooking oil even now gains surreptitious access to the American kitchen only under the guise of packages and labels suggestive of lard. In fact, though several varieties of oil-yielding seeds, such as rape seed, poppy seed, and their oils, have been annually imported into the United States in considerable quantities for special purposes, the popularity of animal fats for all uses to which they are adapted, has, together with other causes, had a tendency to restrict the use of such oils, and thereby not only to place a limit upon their importation, but also to discourage the production of the exclusively oil-yielding seeds upon American soil."

The domestic consumption of all oils of this class amounted in 1860 to less than 9,000,000 gallons. The marvelous advance that has been made in the production of oleaginous seeds and in the consumption of their oils in the United States since that date is illustrated by the fact that at the present time there is annually manufactured from the domestic crops of flaxseed and cottonseed a product of from 160,000,000 to 170,000,000 gallons of oil, three-fourths of which probably enters into home consumption."

Note that only a small part of this oil may have been used as edible oil and that the eating of oil has been a matter of gradual education. Some later information is contained in Bulletin No. 40, Department of Commerce and Labor, Bureau of Census. This is as follows:

"The demand for cotton seed meal as a stock food is rapidly increasing in this country and abroad, and the mills in Great Britain are operated more for the meal than for the oil. Every farmer and dairyman there carries in stock a supply of cake and meal in proportion to the number of cattle, horses and sheep owned. A mixture of cotton seed meal and corn chops make a satisfactory feed stuff for hogs, and the meal is a good egg producer for poultry. Some interest is now felt in the possibility of utilizing cotton seed meal for human food. It is cheaper than flour, and is very nutritious. The confectionery trade has discovered that the kernels of cotton seed when parched make a good substitute for peanuts in the manufacture of peanut brittle.

*Oil.*—The real advancement of the last twenty-five years in the cotton seed oil industry has been made by the oil refinery. While there have been many improvements in the machinery of the crude oil mills, the process is to-day practically what it was a quarter of a century ago, but tremendous strides have been made in the improvement of the refining methods, and the products obtained at this time are quite superior to those formerly produced.

The first process of refining produces from crude cotton seed oil a clear brilliant yellow oil, which, at 50 degrees Centigrade, has a specific gravity of 0.92. Owing to the deterioration of the seed and to inferior methods of manufacture, the first refining of crude oil does not always produce oil of the same grade. This oil, known as "summer yellow" oil, has been classed by the trade as choice, prime, off, and soap oil, the difference in these grades being in the color and flavor. Choice oil is a light, lemon-colored oil, without any appearance of red, and is mild and neutral in flavor. Prime oil is slightly darker in color and sweet in flavor. These two grades are used for edible purposes. The off and soap grades are reddish in color and the flavor is very poor. The quality and amount of oil produced depend largely upon the condition of the seed. The quantity varies from 83 to 35 per cent. of choice and prime oil and from 15 to 65 per cent. of off and soap oil. After being submitted to various processes, such as bleaching, to make it white, and pressing, to extract the stearin, summer yellow oil forms an important basis for a number of different products. With the improvements in refining methods made in the past few years, new uses for cotton seed oil have been developed, among the most important of which is the manufacture of lard compounds, a mixture of hog lard, oleo stearin, and refined cotton seed oil, making a most palatable and economical food. So large a percentage of our home consumption of cotton seed oil is taken by the lard compound makers that the value of the oil varies with the supply of hog lard. The home consumption of cotton seed is larger this year than for previous years, on account of the strong statistical position of the lard market. A very high-class food product, known as "white cottolene," is secured by mixing oleo stearin with specially refined cotton seed oil. It is also used in making salad oils and in the manufacture of the olive oil of commerce.

Cotton seed oil is sometimes used in olive growing countries in "setting" the olives, a quantity of oil being poured over the ripe fruit after it has been placed in vats. This softens the fruit, hastens the flow of oil, and materially increases the yield. Cotton seed oil is also used in packing sardines, in the oleomargarine industry."

It has been suggested to me that experiments be carried on and in this way establish the truth of my theory. Many will doubtless insist upon delaying the acceptance of the idea until this has been done. To these I will call attention to results obtained by feeding cotton seed products to stock. Below is given information contained in Farmer's Bulletin No. 36, U. S. Department of Agriculture.

The average per cent. of oil in cotton seed meal is 13.45. That of the hulls is 2.22. 77 per cent. of the oil in the hull is absorbable.

### Feeding Cotton Seed Products to Farm Stock.

Practical experience has been supplemented by carefully conducted experiments, both in the United States and Europe, with cotton seed, cotton seed hulls, and cotton seed meal as food for cattle, sheep, pigs, horses, and mules, with the result of demonstrating their high feeding value for all kinds of farm stock, with the possible exception of calves and pigs, to which they have frequently proved fatal.

The high feeding value of whole cotton seed has long been recognized, having been fed raw, roasted, steamed, or boiled, to live stock, especially cattle. Almost from the beginning of cotton culture in this country it has been used to some extent as a feeding stuff, but since the introduction of the cotton oil industry the superior feeding quality of the by-product—cotton seed meal—has led to a very general displacement of whole seed by the meal in localities where the latter is easily and cheaply obtained.

The value of cotton seed meal for producing meat, milk, and butter is well established. It is one of the cheapest of the highly nitrogenous feedings stuffs, and is therefore one of the most economical for balancing rations deficient in protein, such as those in which corn is the principal grain. As the analyses show, it is very concentrated and should be fed in comparatively small quantities in connection with a large proportion of coarse feed, such as silage, corn, straw, corn stover, cotton seed hulls, etc., or with good pasturage.

Although milch cows will do well for an indefinite period on cotton seed or cotton-seed meal as the sole grain food, it is better to add a second, such as corn meal or wheat bran, to the ration. If fed to cows in large amounts without proper admixture of other feed stuffs it is likely to injure the quality of the butter as regards flavor and color. It appears, however, to harden the butter and thus enable it to stand shipment better. It has also been found to facilitate very materially the rise of cream by gravity.

Referring to the analyses of cotton seed hulls and meal in the previous pages, we find that neither of them is adapted for use alone as food. The hulls contain a large excess of non-nitrogenous matter and the meal a large excess of protein; each lacks what the other has in abundance. The meal is well adapted by its composition to be fed with the hulls, and the hulls find their proper supplement in the meal. This relation is so evident that the fact that it was not pointed out much sooner is peculiar, although the uninviting character of the hulls as food doubtless had the effect of diverting both scientific and practical investigations from them.

The pratice of fattening steers on a diet made up exclusively of cotton seed hulls and meal commenced about 1883. The business has so grown that it is estimated that probably 400,000 cattle, besides large numbers of sheep, were fattened at and near the oil mills of the South in the season of 1893-94. It is also likely that 100,00 to 150,000 milch cows were fed on rations made up quite largely of cotton seed hulls and meal. The usual ration for feeding cattle is three or four pounds of meal at first, which is gradually increased to six, eight and even ten pounds per head per day, and all the hulls they will eat. The proportions vary from two to six pounds of hulls to one of meal, the most common ration at present probably being four of hulls to one of meal. The feeding is continued from 90 to 120 days. All the information at hand indicates that this practice is both economical and profitable. The diet, apparently, does not injure the health of the animals nor impair the healthfulness of the resulting products, beef, mutton, milk and butter.

The North Carolina Station gives the following rules for the use of cotton seed meal and cotton seed hulls under different conditions:

(1) *For Maintenance.*—Where it is desirable to feed an animal just sufficient to maintain it without loss, the following directions may be followed: Hulls from rather green seed may be fed alone, the particles of seed kernels remaining accidentally with the hulls being counted on for maintenance, or, perhaps, even for slow fattening. Dependence, of course, is placed on the amount of kernels left in the hulls. With well-cleaned hulls, however, some cotton seed meal must be used, depending somewhat on the animal fed. With a cow weighing 950 pounds one pound of meal to every seven pounds of hulls has been shown to maintain the weight and produce about twenty ounces of milk per day. Probably eight or ten pounds of hulls to one pound of meal when fed in quantity (as much as can be eaten clean) will support life and maintain the weight of neat stock.

(2) *For Slow Fattening.*—Rations ranging from seven pounds of hulls to one of meal down to five or four to one may be used, depending on the animals fed and the skill of the feeder. Each animal should be provided with just good growth, and in mature animals it may be counted on to fatten in from 80 to 100 days.

(3) *For Quick Fattening.*—Rations for making good beef quickly may range from four to one down to two to one, or even 1.5 to one, as we have fed steers successfully on the latter ration. For feeding half-fat cattle from 30 or 40 to 60 days, these last rates are well calculated to increase the body weight. But it is doubtless a good plan to heed the German standard and feed the wider ration at the last, in order that more of the digestible food may be fixed as muscular tissue.

(4) *For Milk.*—For the greatest flow of milk we consider it a doubtful practice to feed exclusively on hulls and meal, though both may be prominent articles in the ration. If cotton seed meal is fed in quantities sufficient to support a cow giving a large flow of milk, it may occasion danger to her health, as it certainly does where fed to pigs and calves in like manner. When a cow has passed about four or five months of gestation, and the flow of milk has greatly diminished, she may be put on a ration of hulls and meal, which may be varied from four to one to as much as seven or eight to one of hulls to meal until she has dried off. This will support the cow well. It wonld be well all this time, however, to be feeding once per day some hay, stover, straw, or let her graze part of each day.

For two or three weeks before calving the cow's ration should be changed by substituting a succulent diet or bran for the cotton seed meal. A week before calving, if not already affected by the succulent diet, the cow should be thoroughly purged with Glauber's or Epsom salts, in one pound doses. Care should be exercised to see that the bowels remain loose, if not, repeat the dose at intervals, as needed, until the cow has come to her full yield of milk after calving.

(5) *For Other Stock.*—To other than ruminating animals, the use of either cotton seed hulls or meal is yet of doubtful expedience. Hulls are considered too bulky for horses, but cotton seed meal may often be fed in small quantities to good advantage with the usual wide rations. Its action, however, on the nervous system is yet untried, so far as we are informed, and it would only be safe as a small part of a ration to be used, much as linseed meal or flaxseed is sometimes used. This meal, in small quantities, is not as laxative as linseed meal.

### Effect on the Health of Animals.

Injurious effects of cotton seed products on certain kinds of farm stock have frequently been observed, and their cause has been the subject of many careful investigations, but it is still an open question whether the injurious principle is an original constituent of the cotton seed products or whether it is developed as the result of decomposition before feeding or of a change within the animal's body. The indications are, however, that the chief danger lies in the use of material which has undergone fermentation. All experience goes to show that fresh cotton seed products, especially cotton seed meal, can be safely fed to beef cattle, milch cows, and sheep, although on account of its *extreme richness,* it should be used with care in connection with less concentrated feeds. It may be used with safety in *larger* quantities in *winter feeding* than in *summer feeding.* There is no doubt that its use as a food for young animals, especially pigs and calves, is attended with great danger. Its effect on horses and mules has not as yet been sufficiently studied to warrant conclusions, but a few instances are reported in which it has been fed regularly for long periods with good results."

A careful consideration of cotton seed reveals only one constituent that is incompatible with animal health. The oil has been shown to differ from the normal animal fat and is in part deposited in the animal as cotton seed oil.

Since the preliminary report has been published this statement has been questioned and Dr. H. W. Wiley, Chief of Bureau of Chemistry, Department of Agriculture has been quoted in an advertisement of cotton seed products as having stated that the oil is changed during digestion.

Confirmation of my statement may be found in any standard work on chemistry of oils and fats. Much has been said of the high digestibility of edible cotton seed oil products. It is true that they are easily digested, but it does not follow that they are wholesome and compatible with good health. In the Daily Consular and Trade Reports No. 3485, Page 14, will be found a statement by Dr. Wiley to the effect that no work by his department on the question of the wholesomeness of cotton seed oil products has been done. This statement was made in response to an effort to combat the charge of M. Plinchon, a member of the French tariff commission, who declared publicly before that commission that cotton seed oil was injurious to health and destroyed the abdominal tissues.

Note in the above that milch cows of all farm stock will do well for an indefinite period on cotton seed or cotton seed meal. It is significant to know that butter from these cows will respond to the test for cotton seed oil, showing that at least some of the fat is eliminated and not deposited in the body. The fat so eliminated is probably a large per cent. of that ingested, as cows during lactation do not usually take on fat. Note the caution relative to pigs and calves, or young animals, and the results if fed during the summer. The food recommended for a cow weighing 950 pounds is one pound of meal to seven pounds of hulls. This amount of meal and hulls contains .2423 pounds or about four ounces of fat per feed. Two daily feedings represent about a half-pound of fat. In the same proportion a man weighing 160 pounds should receive about 5-12 of an ounce of fat per meal, provided he has some method of elimination to take the place of lactation. Also note that the above report recognized the fat as the injurious constituent. I wish to submit that the above examples are conclusive evidence that the oil is the injurious agent. The pigs and calves accumulate the fat and die as the result. The cow eliminates fat and is protected.

It is stated that the usual period for fattening beef cattle is 90 to 120 days, which is too short a time for the damage to become evident. Also, precautions are probably taken to limit exercise, and facilitate the fattening process. In limiting exercise oxidation is thereby reduced. You will also note that the practice of fattening steers on this exclusive diet commenced about 1833. Observe then that we have several sources for getting cotton seed oil in our bodies besides that consumed as a substitute for lard. From the beef fat, butter, cooking oils. compound lard and salad oil we could easily overreach the danger line shown to exist in milch cows. Without her protection it is reasonable to suppose that a human being would exhibit serious damage from consuming a food shown to produce disease and death in other animals. Besides the evidence of the deleterious effects shown to be produced by the above, I wish to emphasize the fact that cases of pellagra from whose diet cotton seed oil has been excluded there has been not one recurrence of the disease. Also cases who have continued to use cotton seed oil have continued to have pellagra. I have had the opportunity of investigating 200 cases of Pellagra and all have eaten cotton seed oil.

In order to show that this fact is of signfiicance, we began a record to determine what per cent. of our cases not suffering with the disease fulfilled this condition. The results were, 48 per cent using cotton seed oil. Eighty per cent. of these have begun the use of it in the last three years. Many of the users presented marked symptoms of "Pellagra sine Pellagra." There is much evidence of an experimental nature that I hope to present at a future date. I believe that enough has been given to the medical profession to justify a withdrawal of cotton seed oil products from the diet of cases of Pellagra. This is the only experiment that can practically demonstrate the truth or fallacy of this theory.

---

### SURGICAL SUGGESTIONS.

In diarrhoea with bloody stools, do not overlook the possibility of rectal polypi being present.

In cases of chronic suppurative disease of the middle ear, with perforation of the drum, when the discharge cannot be arrested by appropriate treatment, there is good reason to suspect tuberculosis as the cause.

In examining for joint disease in children, the element of fear must always be considered. A frightened child will manifest more or less muscular resistance and tension, which may deceive the examiner as to the true existing condition. Therefore it is well to divert the little patient's attention by letting him think that the various manipulations are only a game for his amusement.

In amputations on diabetic subjects it is advisable to dispense with the torniquet whenever possible, owing to the effect of the constriction upon the poorly nourished tissues. For the same reason it is important to avoid any forcible manipulations.

—American Journal of Surgery.

